# Coverage, compliance and factors associated with utilization of iron supplementation during pregnancy in eight rural districts of Ethiopia: a cross-sectional study

**DOI:** 10.1186/1471-2458-14-607

**Published:** 2014-06-14

**Authors:** Samson Gebremedhin, Aregash Samuel, Girma Mamo, Tibebu Moges, Tsehai Assefa

**Affiliations:** 1 School of Public and Environmental Health, Hawassa University, Hawassa, Ethiopia; 2 Ethiopian Public Health Institute (EPHI), Addis Ababa, Ethiopia; 3 Micronutrient Initiative (MI), Addis Ababa, Ethiopia

**Keywords:** Iron supplementation, Maternal anemia, Compliance

## Abstract

**Background:**

Despite the efforts to reduce iron deficiency during pregnancy, information on the coverage and factors associated with utilization of iron supplements is lacking. The study is intended to assess the coverage, compliance and factors associated with the use of prenatal iron supplements in eight rural districts of Ethiopia.

**Methods:**

The study comprised two independent surveys conducted among pregnant women (n = 414) and women who gave birth in the preceding year of the survey (n = 1573). In both cases, respondents were selected using multistage sampling technique and data were collected via structured questionnaire. Predictors of iron supplement utilization (ranked categories of number of prenatal supplements taken) were identified using ordinal logistic regression. The outputs of the analysis are given using adjusted Odds Ratio (OR) with 95% Confidence Interval (CI).

**Results:**

Among women who gave birth in the preceding year, 35.4% (95% CI: 31.3-39.5) were given/prescribed prenatal iron supplement during the index pregnancy and only 3.5% were supplemented for the recommended 91 or more days. Compared to women who had 4 or more ANC visits, those with 0, 1, 2 and 3 visits had 0.04, 0.33, 0.50 and 0.60 times less odds of iron supplement utilization, respectively. Women lacking comprehensive knowledge of anemia (OR = 0. 75 (95% CI: 0.57-0.97)) and those who weren’t informed about the importance of iron supplementation during the pregnancy (OR = 0. 05 (95% CI: 0.04-0.07)) had significantly lower utilization. On the other hand, in pregnant women the prevalence of anemia was 33.2%. Among pregnant women who were given/prescribed supplements, the average level of compliance was 74.9% and about 25.1% had less than 70% adherence. The leading reported reasons for non-adherence were side-effects (63.3%) and forgetfulness (16.7%).

**Conclusion:**

Promoting early and frequent ANC, enhancing the quality of ANC counseling and promoting the knowledge of women on anemia are essential strategies for improving the utilization of iron supplements.

## Background

Anemia is a global public health problem affecting two billion people worldwide [1]. Particularly pregnant women and preschool-age children take its disproportionate burden. Globally, almost half of all preschool children (47.4%) and pregnant women (41.8%) and close to one-third of non-pregnant women (30.2%) are anemic [1,2]. Though anemia has multifaceted causes, half of its burden is attributed to Iron Deficiency (ID) [2].

Several studies documented the consequences of maternal anemia including increased risk of maternal death, low birth weight and preterm birth [3]. According to the World Health Organization (WHO), 3.7% of maternal mortality in Africa is directly attributed to anemia [4]. Besides ID is an underlying cause for 22% of maternal deaths worldwide [5]. A meta-analysis indicated that the risk of maternal death can be reduced by 20% for each 1 g/dl increase in population mean hemoglobin level [5].

In Ethiopia many studies witnessed the public health significance of maternal anemia [6-10]. Accordingly, in confirmation of the WHO recommendation [11], the national guideline for control and prevention of micronutrient deficiencies highlights the need of daily iron supplementation for at least 6 months during pregnancy and 3 months postpartum [12]. The National Nutrition Strategy (NNS) also set a key target of increasing the proportion of mothers who get iron supplementation for more than 90 days during pregnancy and the postpartum period to 50% by 2015 [13].

According to Ethiopia Demographic Health Survey (EDHS) 2011, the coverage of iron supplementation is disappointingly low as only 17.3% of women took the supplement during their recent pregnancy in the preceding 5 years and only 0.4% were supplemented for 90 or more days [6]. Despite the assumption that iron supplementation is an integral part of Antenatal Care (ANC), only 37% of women who had ANC received iron supplements [6].

Thus the purpose of the current study is to assess the coverage, compliance and factors associated with utilization of iron supplementation during pregnancy in eight selected rural districts of Ethiopia. The study also appraised prevalence of anemia during pregnancy and the association between duration of iron supplementation and maternal hemoglobin concentration. The study is focused on iron supplementation – rather than iron-folate supplementation – as the availability of the latter was limited throughout the health institutions of the districts in the reference period of the study.

## Methods

### Study setting

The study was conducted from February to March 2012 in eight rural *woredas* of the four major regions of Ethiopia (Tigray, Amhara, Oromya and Southern Nations Nationalities and Peoples (SNNP) regions). A *woreda* –hereafter referred to as district – is an administrative division typically composed of 20–40 *kebeles. Kebele* is the smallest administrative unit with an average population of 5000. The districts included in the study were: Almata and Enderta from Tigray region, Menze Mama and Menze Gera from Amhara region, Ada Berga and Meta Robi from Oromya region, Sankura and Meskan from SNNP region. The districs were selected from their respective regions due to their relative accessability to the center of the country. In Ethiopia prenatal iron supplementation is the integral part of ANC and its provided free of charge; nevertheless, the program suffers from supply and logistic challenges.

### Study design

The study embraced two distinct community based surveys carried out simultaneously in the eight districts. The first survey – conducted among women who gave birth in the preceding one year – was designed to assess the level and factors associated with the utilization of iron supplements during pregnancy. The other survey – conducted among pregnant women – was intended to appraise pregnant women’s compliance with iron supplementation and its association with hemoglobin concentration.

### Sample size

The sample size for the study conducted among non-pregnant women was calculated as 1573 using single proportion formula. The inputs of the computation were: 95% confidence level, 2% margin of error, expected iron supplementation coverage of 17% [6], design effect of 1.5 and non-response rate of 10%. Further, based on the power calculation formula [14] designed for ordinal logistic regression analysis, the available sample size is judged to be optimal for identifying the factors associated with utilization of iron supplementation with 95% confidence level and 90% power and small effect size (Odds Ratio (OR) of 1.5) to be detected.

The sample size for the survey among pregnant women was computed as 445 using single mean formula. The computation was made with 95% confidence level, 0.20 Standard Deviation (SD) for mean adherence [15], 0.05 margin of error, design effect of 1.5 and 10% non-response rate. Further, corrections were made for finite population size and expected coverage of iron supplementation. In both of the surveys, the sample sizes were equally distributed to the eight districts and the 24 *kebeles* included in the study.

### Sampling technique

In both of the surveys multistage sampling technique was applied to select the study participants. Initially from each district 3 rural *kebeles* were selected using simple random sampling technique. The sampling frames for the secondary sampling units (pregnant women and women who gave birth in the preceding 12 months) were developed by mobilizing local Community Health Promoters (CHPs) working at sub-*kebele* level. Ultimately, participants were selected using simple random sampling technique.

### Data collection method

In the surveys, information was gathered using pre-tested structured questionnaires. The tools were finalized in Amharic and administered in the local languages. The data were gathered by 24 trained female data collectors. For the non-pregnant women, interviews were made at home; however, the pregnant women were invited to the nearby health posts so that blood samples can easily be collected. Nearly the entire (93%) pregnant women invited to the health post took part in the study.

In order to assess the knowledge of pregnant women on anemia, different local names of anemia in the five local languages spoken in the study districts were used. The local names were identified ahead through conducting group discussions with local mothers and in-depth interviews with Health Extension Workers (HEWs) in each of the eight districts.

Among pregnant women, hemoglobin concentration was determined using a drop of capillary blood via HemoCue Hb 301 following standard procedures. Hemoglobin values were adjusted for altitude according to the standard of the Centers for Disease Prevention and Control (CDC) [16]. Based on hemoglobin concentration, anemia status was classified as normal, mild, moderate and severe using trimester specific cutoff points.

### Data management and analyses

The data were entered into SPSS 20.0 and exported to STATA/SE 11.0 for analysis. Considering the complex sampling technique used in the study, descriptive analyses were made using the survey (svy) command after specifying the appropriate sampling design, probability weights and sampling fractions.

Wealth index was computed as a composite indicator of living standard using Principal Component Analysis (PCA). The data reduction was made based on 25 input variables related to ownership of selected household assets, size of agricultural land, quantity of livestock and materials used for housing construction. Ultimately, a principal component was generated and it was divided into 5 equal wealth quintiles (poorest, poorer, middle, richer, and richest).

Factors associated with the utilization of iron supplementation were identified using ordinal logistic regression. The response variable (duration of iron supplementation) was categorized into five ranks: no supplementation, 1–30, 31–60, 61–90 and more than 90 days of supplementation. Categorical ranks, rather than actual days of supplementation, were preferred as the latter is more liable to recall errors.

For the logistic model, eleven independent variables were selected based on review of relevant literatures. The variables were: household wealth index, maternal literacy, husband’s literacy, birth order, maternal age, distance from the nearest health facility, frequency of ANC, receiving information about the importance of iron supplementation during the pregnancy, comprehensive knowledge of anemia, previous experience of life threatening pregnancy complication and whether the pregnancy was wanted or not.

At the outset, the association of every independent variable with the response variable was evaluated using bivariate analysis. Subsequently, statistically significant variables (*P < 0.05*) were considered in the multivariate model. The major assumption of ordinal logistic regression – homogeneity of OR – was checked using the test of parallel lines. The fitness of the mode was assessed via Pearson and Deviance chi-square tests. The association between duration of iron supplementation and hemoglobin concentration was evaluated using linear regression analysis.

### Ethical considerations

The study was conducted in confirmation of ethical guidelines for research involving human subjects. Ethical clearance was obtained from the scientific and ethical review office of Ethiopian Public Health Institute (EPHI). The data were collected after taking informed verbal consent form the study participants. All pregnant women who were not on ANC were linked with the health post so that they can start the service. The transportation costs of the pregnant women who traveled to the nearby health posts for the study were covered by the project.

## Results

### Survey among women who gave birth in the last 12 months

#### Socio-demographic information

Data were successfully gathered from 1563 women who gave birth in the preceding 12 months with a response rate of 97.1%. The mean age of the respondents was 27.6 (±6.1) years and nearly three-quarters (74.9%) were between 20–34 years. The median number of children ever born (CEB) was 4. Nearly two-third (67.3%) of the respondents were illiterates and more than half (56.9%) identified themselves as housewives or farmers (Table 1).

**Table 1:** Socio-demographic characteristics of the study participants, eight selected rural districts of Ethiopia, March 2012

**Background characteristics**	**Frequency**	**Percent**
Maternal age in years (n = 1559)		
15-19	119	7.6
20 -34	1167	74.9
≥ 35	273	17.5
Children ever born (n = 1552)		
1	272	17.5
2-4	763	49.1
≥ 5	517	33.3
Educational status (n = 1563)		
Can't read or write	1052	67.3
Informal education	24	1.5
Primary education	363	23.2
Secondary or tertiary education	124	7.9
Marital status (n = 1561)		
Married/living together	1431	91.7
Divorced/separated	91	5.8
Never married	29	1.9
Widowed	10	0.6
Occupation (n = 1563)		
House wife/farmer	889	56.9
Student	467	29.9
Petty trade	161	10.3
Others	46	3.0

#### Awareness about maternal anemia

Near to three-fourth (72.0%) of the respondents had ever heard of anemia. About 67.9% and 57.6% managed to tell at least one of the major symptoms and causes of anemia, respectively. Failure to take iron supplements during pregnancy and inadequate dietary iron intake were recognized as causes by 42.3% and 25.9% of the respondents. Two-third (62.5%) mentioned at least one of the consequences of maternal anemia. Increased risks of maternal death (42.1%), serious maternal illness (40.5%) and fetal death (16.2%) were the frequently mentioned ones.

Comprehensive knowledge of anemia was measured using a composite index constructed based on multiple indicators. Respondents who were aware of anemia and know at least one of its major causes, symptoms and consequences during pregnancy were considered to have comprehensive knowledge. Nearly half (51.4%) of the respondents had comprehensive knowledge of anemia.

About 38.4% claimed that during the recent pregnancy, they had been informed about the significance of iron supplementation. The principal sources of information were HEWs (19.9%) and health professionals (19.8%). Family/neighbors (2.1%) and CHPs (1.5%) were seldom mentioned. Very few of the respondents were aware that the supplement has to be taken on a daily basis (37.1%) and it should be taken throughout the pregnancy (13.0%).

#### Coverage of iron supplementation during pregnancy

About 35.4% (95% CI: 31.3-39.5) of the women were given/prescribed iron supplements during the pregnancy they had in the last 12 months. Amongst women who had at least one ANC visit, less than half (46.8%) received iron supplements. Proportions who were given/prescribed iron among those who had 1, 2, 3 and 4 or more ANC visits were 35.5, 39.8, 49.3 and 59.2%, respectively.

**Figure 1:**
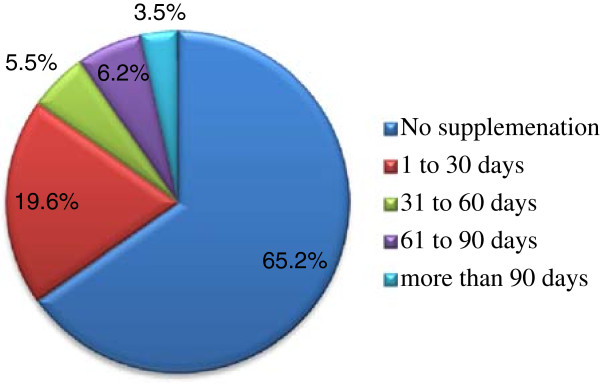
Number of days of iron supplementation during the recent pregnancy in the last 12 months, eight selected rural districts of Ethiopia, March 2012.

Of the entire respondents 34.7% (95% CI: 30.7-38.7) took the supplement at least once during the index pregnancy. The mean (±SD) gestational age at the beginning of the supplementation was 5.6 (±1.7) months and the median total duration of the supplementation was only 30 days. In overall, 19.6%, 5.5% and 6.2% took the supplements for 1–30, 31–60 and 61–90 days, consecutively. In contrast to the national recommendation [13], fewer than one-in-twenty women (3.5%) took the supplements for more than 90 days (Figure 1).

#### Factors affecting utilization of prenatal iron supplementation

As discussed in the methods section, the level of iron supplementation utilization was subdivided into five ordinal categories and modeled using ordinal logistic regression. Eleven factors were considered as explanatory variables. In the univariate analysis, maternal age and birth order were not significant predictors thus the multivariate model was built on the remaining nine variables. Ultimately, maternal literacy, wealth index, frequency of ANC, comprehensive knowledge of anemia and receiving information about the importance of iron supplementation during pregnancy were the significant predictors.

Compared to women who had the recommended 4 or more ANC visits, those with 0, 1, 2 and 3 visits had 0.04 (95% CI: 0.02-0.07), 0.33 (95% CI: 0.19-0.58), 0.50 (95% CI: 0.31-0.79) and 0.60 (95% CI: 0.44-0.80) times less odds of iron utilization, respectively. Women who did not have comprehensive knowledge of anemia and those who were not informed about the importance of iron supplementation during the pregnancy had poor utilization with an adjusted OR of 0.75 (95% CI: 0.57-0.97) and 0.05 (95% CI: 0.04-0.07), respectively. Unexpectedly, illiterates and mothers from the poorest quintile had significantly better utilization compared to their counterparts (Table 2).

**Table 2:** Factors associated with utilization of iron supplementation during pregnancy, eight selected rural districts of Ethiopia, March 2012

**Variables**	**Crude OR**	**Adjusted OR**
Maternal age in years		
15-19	0.84 (0.54-1.30)	-
20-34	1.07 (0.83-1.40)	-
35-49	1.00^r^	-
Maternal education		
Illiterate	1.26 (1.02-1.56)*	1.61 (1.22-2.13)*
Literate	1.00^r^	1.00^r^
Paternal education		
Illiterate	1.30 (1.06-1.61)*	1.13 (0.87-1.49)
Literate	1.00^r^	1.00^r^
Household wealth index		
Poorest	1.50 (1.11-2.04)*	1.80 (1.22-2.64)*
Poorer	0.91 (0.66-1.25)	0.95 (0.64-1.41)
Middle	0.86 (0.26-1.18)	1.15 (0.78-1.70)
Richer	0.91 (0.66-1.24)	0.91 (0.62-1.34)
Richest	1.00^r^	1.00^r^
Birth order		
1	1.00 (0.75-1.33)	-
2-4	1.07 (0.86-1.34)	-
5 or more	1.00^r^	-
Two-way distance from the nearest health facility		
Less than 30 minutes	1.00^r^	1.00^r^
30 minutes to one hour	0.91 (0.67-1.24)	1.22 (0.85-1.75)
More than one hour	0.63 (0.49-0.82)*	0.86 (0.63-1.17)
Frequency of ANC visits during pregnancy		
0	0.03 (0.02-0.05)*	0.04 (0.02-0.07)*
1	0.37 (0.23-0.59)*	0.33 (0.19-0.58)*
2	0.44 (0.29-0.65)*	0.50 (0.31-0.79)*
3	0.63 (0.49-0.81)*	0.60 (0.44-0.80)*
4 or more	1.00^r^	1.00^r^
Experience of serious pregnancy complication before		
Yes	1.00^r^	1.00^r^
No	0.77 (0.62-0.96)*	0.82 (0.63-1.07)
Comprehensive knowledge of anemia		
Yes	1.00^r^	1.00^r^
No	0.35 (0.28-0.43)*	0.75 (0.57-0.97)*
Information about importance iron supplementation during the pregnancy		
Yes	1.00^r^	1.00^r^
No	0.04 (0.03-0.05)*	0.05 (0.04-0.07)*
Pregnancy wanted		
Yes	1.00^r^	1.00^r^
No	0.74 (0.59-0.91)*	1.11 (0.85-1.44)

^*^Significant association at 95% confidence level, ^r^Reference group.

### Survey among currently pregnant women

#### Prevalence of anemia in pregnancy

**Figure 2:**
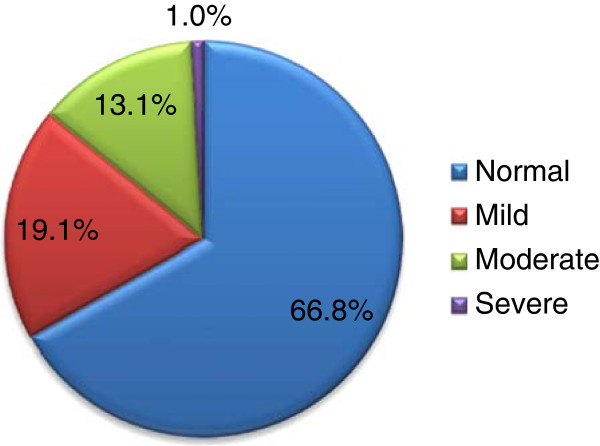
Prevalence of anemia during pregnancy, eight selected rural districts of Ethiopia, March 2012.

Of the recruited pregnant women 414 (93.0%) took part in the study. About two-third (65.0%) were illiterates and the mean age of the respondents was 26.4 (±5.7) years. The median gravidity was 4 and ranged from 1 to 12. Regarding gestational age, 1.5%, 65.1% and 33.3% were in their first, second and third trimesters, respectively (Data not shown).The mean hemoglobin concentration adjusted for altitude was 11.5 (±1.6) g/dl and ranged from 4.1-16.3 g/dl. In overall, the prevalence of any form of anemia was 33.2% and the magnitudes of mild, moderate and severe anemia were 19.1, 13.1 and 1.0%, respectively (Figure 2).

#### Association between iron supplementation and hemoglobin

**Figure 3:**
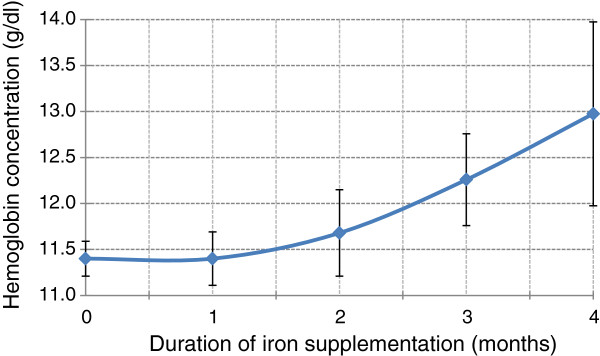
Mean maternal hemoglobin concentration (with 95% confidence interval) across various durations of iron supplementation, eight selected rural districts of Ethiopia, March 2012.

During the survey, 66.2% of the pregnant women were not on iron supplementation; whereas, 19.0%, 7.2%, 6.4% and 1.1% took the supplements for 1–30, 31–60, 61–90 and more than 90 days, respectively. The mean hemoglobin (±SD) concentrations in women who took the supplements for 0, 1, 2, 3, and 4 months were 11.4 (±1.6), 11.4 (±1.7), 11.7 (±1.5), 12.3 (±1.6) and 13.0 (±0.7) g/dl, respectively. Despite the wide confidence intervals seen in the last 3 categories due to limited sample sizes, hemoglobin concentration showed a linear increment after the first month of supplementation (Figure 3). In a linear regression model adjusted for gestational trimester, every month of supplementation was associated with a significant 0.23 g/dl increment in hemoglobin (*t = 2.692. P = 0.037)*.

#### Compliance to prenatal iron supplementation

Among 115 mothers who were given/prescribed iron supplements during the pregnancy, the level of compliance was assessed based on the reported number of doses taken in the preceding 15 days. The overall compliance was 74.9%. It means, the respondents on supplementation on average took only three-fourth of the recommended doses. Furthermore, 32.6% and 25.1% had less than 90% and 70% levels of compliance (Table 3). Amongst women who missed 2 or more doses, the leading underlying reasons were side-effects (63.3%), forgetfulness (16.7%), running out of supplements (10.0%) and alleviation of the symptoms of anemia (6.7%).

**Table 3:** Level of compliance with iron supplementation in the preceding 15 days of the survey, selected eight rural districts of Ethiopia, March 2012

**Number of iron supplements taken in the preceding 15 days**	**Corresponding level of compliance**	**Percent**
14-15	> 90%	67.4
11-13	70-90%	7.5
0-10	< 70%	25.1

## Discussion

According to EDHS 2011, only 17.3% of women took iron supplements during the recent pregnancy they had within 5 years [6]. However, our study reported relatively higher level of utilization (34.7%) probably due to the reason that it was conducted in comparatively accessible districts of the four better off regions of the country. Furthermore, the longer reference period used in the DHS might have made it less sensitive to the very recent national level interventions targeted to move up iron supplementation coverage.

The NNS of Ethiopia envisages increasing the proportion of mothers who get iron supplementation for more than 90 days during pregnancy and the postpartum period to 50% [13]. Yet in the study districts, only 3.5% of the women had such level of utilization during pregnancy and the practice was non-existent in the postpartum period. Besides, most women started the supplement in the late second or third trimester. Taking into account the relative accessibility of the study districts, a gloomier national level figure can be anticipated. This can be taken as strong evidence that extraordinary interventions are needed to materialize the target.

Existing studies applied assorted criteria to define compliance with iron supplementation; hence, comparison among them is not viable. Nonetheless, the reported level of compliance (75%) might have been overestimated as it is measured based on the self-reporting method. A reasonable number of studies witnessed that self-reporting overestimates compliance as compared with pill count or biochemical method. According to a study in Indonesia [17], in 43% of the pregnant women who have claimed to have taken all iron tablets, the same can’t be confirmed using stool tests. Studies in Egypt [18] and Philippines [15] also found significantly higher level of compliance as compared with the pill count method.

ANC is the vital route for delivery of iron supplementation and reinforcement of adherence. Consequently, the association observed between the frequency of ANC and level of iron utilization is an expected one. Several studies have also documented the same [18-20]. In contrast, we found nearly half of the women who had ANC were not given iron supplements. This can be due to shortage of iron supply and lack of effective logistic system to distribute the supply. A study in Tanzania also reported, among women who had 2 or more ANC visits, the coverage of iron supplementation was only 15 to 21% [19].

Several studies found better iron supplement utilization among socio-economically empowered women [19,21-23]. Yet in the current study the opposite is reported. The unexpected finding can be due to a couple of reasons. The well-off mothers might consider themselves less susceptible to anemia as a result they might have poor compliance. Or the expected higher prevalence of anemia among economically disadvantaged women might have compelled them to adhere better. According to a study in Saudi Arabia, 10% of the women with low compliance miss the supplements believing that they had adequate diet [22].

WHO had recommended a criterion to evaluate the public health significance of anemia based on its prevalence in pregnant women [24]. The prevalence reported in the current study (33%) falls within the category of 20-40% indicative of moderate public health significance. Other large scale surveys in Ethiopia concluded the same. The recent two DHS surveys conducted in 2011 [6] and 2005 [7] and another large-scale survey carried out in 2005 [9] reported 22%, 27% and 31% prevalences among pregnant women, respectively.

Limited proportion of the women (51.4%) had comprehensive knowledge of anemia and only 38.4% were informed about the significance of iron supplementation during the pregnancy. However, both of the variables turned out to be strong predictors of iron supplement utilization. The finding indicates that enhancing the awareness of mothers can substantially improve the utilization of iron supplementation. Previous studies conducted in Cambodia [20] and Sweden [25] also came up with parallel findings.

Side-effect is frequently considered as a major obstacle to compliance. According to studies in Saudi Arabia [22], Senegal [26] and India [27], it was reported as a reason for missing doses by 40.2%, 27.0% and 25.4% of the pregnant women with low adherence, respectively. Studies conducted in Philippines [15], Bangladesh [28] and Vietnam [29] also concluded likewise. In the current study, a relatively higher proportion of women with low compliance (63.3%) reported side-effect as the reason for non-adherence. One possible explanation for the elevated figure can be the fact that nearly all (94%) of the pregnant women were not informed about potential side-effects of iron supplements in advance. Appropriate orientation is known to raise psychological tolerance to side-effects [30].

The study is the first attempt in Ethiopia to understand factors influencing the utilization of prenatal iron supplement. The study sampled respondents from eight districts across the four major regions of the country and included reasonably large sample size. Besides, it complemented two different surveys. However, the following limitations need to be considered while interpreting the findings of the study. Recall error regarding the number of supplements taken in the prior year is possible and it might have affected the precision of the study. Further, as relatively accessible districts are selected, selection bias is likely and the supplementation coverage might have been over estimated; consequently, the findings cannot be generalized beyond the study districts. In addition, pregnant women in the first trimester were largely under represented (1.5%) due to various reasons (the study only used presumptive symptoms of pregnancy to recruit the study subjects and there is common culture of not revealing pregnancy status in public until it becomes obvious). This might have overestimated the prevalence of anemia and utilization of iron supplements.

## Conclusion

The coverage of prenatal iron supplementation is unsatisfactory as only 35.4% of the women who gave birth in the preceding year were given/prescribed iron tablets. Factors adversely associated with the utilization of the supplements were having no or too few ANC visits, lack of comprehensive knowledge of anemia and unable to get advice about the importance of the supplement during the pregnancy. Of pregnant women on iron supplementation, the average level of compliance was 74.9%. Amongst women with low adherence, the leading underlying reasons were side-effects and forgetfulness.

Promoting the benefits of early and frequent ANC, enhancing the quality of ANC counseling including guidance on management of side-effects; and promoting the knowledge of women on anemia are essential elements for improving the utilization of the service. Local CHPs should also be strongly involved in the promotion of prenatal iron supplementation.

## Competing interests

The authors declare that they have no competing interests.

## Authors’ contribution

SG drafted the manuscript, performed the statistical analysis and participated in the supervision of the fieldwork: AS conceived, designed and coordinated the study: GM, TM and TA participated in the designing of the study, supervision of the fieldwork and analysis of the data. All authors read and approved the final manuscript.

## Authors’ information

SG: Assistant professor of public health, Hawassa University, Ethiopia.

AS: Associate researcher of food science and nutrition, EPHI, Ethiopia.

GM: Senior program officer of maternal neonatal health and nutrition, Micronutrient Initiative (MI), Ethiopia.

TM: Assistant researcher of food Science and nutrition, EPHI, Ethiopia.

TA: Associate researcher of food Science and nutrition, EPHI, Ethiopia.

## Pre-publication history

The pre-publication history for this paper can be accessed here:

http://www.biomedcentral.com/1471-2458/14/607/prepub
